# Pressurized liquid extraction followed by high-performance liquid chromatography for determination of beta-ecdysone extracted from *Pfaffia glomerata* (Spreng.) Pedersen

**DOI:** 10.1038/s41598-024-64077-6

**Published:** 2024-07-11

**Authors:** Charlini Balastreri Dorta de Oliveira, Lúcio Cardozo Filho, João Carlos Palazzo de Mello, Osvaldo Valarini Júnior, Giselle Nathaly Calaça, Stella Alonso Rocha, Francielle Sato, Otávio Akira Sakai

**Affiliations:** 1 Program in Sustainability, Federal Institute of Paraná, Rodovia PR 323-km 310, Pq. Industrial, Umuarama, PR 87507-014 Brazil; 2 Chemical Engineering, State University of Maringá, Av. Colombo, 5790, Bloco D-90, Maringá, PR 87020-900 Brazil; 3 Department of Pharmacy and Pharmacology, State University of Maringá-UEM, Av. Colombo, 5790, Maringá, PR 87020-900 Brazil; 4 Department of Food and Chemical Engineering, Federal Technological University of Paraná, Via Rosalina Maria dos Santos, 1233, Vila Carolo, Campo Mourão, PR 87301-899 Brazil; 5 Federal Institute of Paraná, Campus Irati, R. Pedro Koppe, 100-Vila São João, Irati, PR 84507-302 Brazil; 6 Department of Physics, State University of Maringá (UEM), Av. Colombo 5790, Maringá, PR 87020-900 Brazil

**Keywords:** Beta-ecdysone, Sustainable, Chemical compounds, Ginseng brasileiro, Chemical engineering, Biotechnology, Sustainability

## Abstract

*Pfaffia glomerata* (Spreng.) Pedersen has among its main bioactive compounds saponins, with the phytoestroid β-ecdysone as its chemical marker. In this study, pressurized liquid extraction (PLE), a green extraction technique used to obtain bioactive compounds from plants, was employed to extract beta-ecdysone from P. glomerata leaves, stems, and roots. The 2^2^ factorial design was used with the variables temperature (333 K and 353 K) and flow rate (1.5 and 2 mL min^−1^), pressure (300 Bar), time (60 min), and solvent [ethanol and distilled water (70:30 (v/v)] were kept constant for all parts of the plant. The results of experimental responses demonstrated that the factors temperature and flow rate significantly interfere with the yields of leaf (0.499%), root (0.65%) and stem (0.764%) extracts. The latter presented presents the highest yield compared to the other parts of the plant. HPLC results showed the presence of beta-ecdysone in all parts of the plant with concentrations of β-ecdysone 86.82, 76.53 and 195.86 mg L^−1^ to leaf, root and stem, respectively. FT Raman results exhibited typical peaks of beta-ecdysone, such as 3310 cm^−1^, 1654 cm^−1^, and 1073 cm^−1^ for all plant parts. Another interesting result was the presence of the peak at 1460 cm^−1^ in the PLE root extract can be associated with selenium. This foundational knowledge confirms that the PLE extraction process was efficient in obtaining the chemical marker of *Pfaffia glomerata* in all plant parts.

## Introduction

The plants of the genus Pfaffia, species *Pfaffia glomerata* (Spreng.) Pedersen belongs to the *Amaranthaceae* family, and this family comprises about 170 genera and 2000 species. Pfaffia *glomerata* known as Brazilian ginseng or “paratudo” is found in tropical regions, mainly in river floodplains due to the amount of humidity^1^. In Brazil, we can find P. *glomerata* in the islands and floodplains from Paraná River- Querência do Norte, state of Paraná, Brazil. The mains active constituents of the roots of P. *glomerata* are saponins, identified by several authors in studies already carried out, with the phytosteroid β-ecdysone being the main saponin present in the roots of P. *glomerata*^2,3^. Researchers have identified other chemical compounds (Pfaffianol A, Pfaffiaglycosides A, B, C, D, E; Pterosterone, Pfaffoside C, Ecdisterone, Aquebonoic acid, GlcA: β-O-glucopyranosiduronic acid, Glc≡β-O-glucopyranosyl, quercetin-3-O-glucoside, kaempferol-3-O-glucoside and kaempferol-3-O-(6-pcoumaroyl)-glucoside, oleanane-type triterpenoid saponins such as ginsenoside Ro and chikusetsusaponin IV, oleanonic acid and gluconic acid) being that the major compound found was β-ecdysone^4–6^. β-ecdysone is the chemical compound identified as 2β, 3β, 14α, 20β, 22,25-hexahydroxy-7-colesten-6-one and known worldwide for its CAS number, 8047-15-2^7–9^. Different clean extraction techniques use the principles of green chemistry^10^ such as ultrasound, microwaves, and supercritical fluid to obtain bioactive compounds, mainly beta-ecdysone, with several applications in the food industry and pharmaceutical^1^.

Extraction by pressurized liquid extraction (PLE) is a sustainable and environmentally friendly technology that uses liquid solvents in their subcritical state with controlled temperature and pressure. Then it usually manages to extract a maximum of compounds in a short period, in addition to obtaining chemical compounds that traditional methodologies. PLE extraction has been applied to obtain natural compounds from plant matrices^11^.

This study proposes to evaluate the presence and quantification of 20-Hydroxyecdysterone (20E) in extracts from different parts (roots, stems, and leaves) of P. *glomerata*, obtained by PLE, using high-performance liquid chromatography and the use Raman spectroscopy to identification compounds chemistry.

## Results and discussion

The conditions of experimental design methodology (Table 4) was used to obtain the yields of PLE described at Table 1. The results showed the yield of dry extract from each part of the ginseng plant, and the percentage of β-ecdysone in the dry extract (%EMSβ). The %EMSβ was obtained by multiplying PLE Extraction (w/w) (Table 2) by the Yield PLE (%).

**Table 1:** Experimental and calculated for PLE differents conditions, (×1) temperature (×2) flow rate.

Run	Yield PLE (%)			PLE extraction (w/w)		
				Leaves	Stem	Roots
	Leaves	Stem	Roots	0.0177	0.0392	0.0153
				%EMSβ	%EMSβ	%EMSβ
R1	13.54 ± 0.85	20.11 ± 0.43	36.34 ± 0.60	0.42 ± 0.03	0.65 ± 0.07	0.51 ± 0.01
R2	28.12 ± 0.51	20.91 ± 0.39	39.48 ± 0.34	0.50 ± 0.02	0.82 ± 0.06	0.60 ± 0.08
R3	28.64 ± 0.28	17.82 ± 0.25	50.52 ± 0.55	0.51 ± 0.01	0.69 ± 0.04	0.70 ± 0.01
R4	33.42 ± 0.98	21.88 ± 0.49	51.29 ± 0.29	0.59 ± 0.03	0.88 ± 0.08	0.78 ± 0.01

**Table 2:** Significance test, standard-error and the respective confidence interval of the %EMSβ.

	%EMSβ—leaves				%EMSβ—stem				%EMSβ—roots			
	Effects	p-value	A	Confidence interval	Effects	p-value	a	Confidence interval	Effects	p-value	a	Confidence interval
A	0.499	0.004	0.003	0.457–0.540	0.764	0.003	0.003	0.760–0.769	0.650	0.002	0.002	0.619–0.679
*x*_1_	0.091	0.004	0.006	0.007–0.174	0.181	0.001	0.006	0.172–0.183	0.089	0.033	0.004	0.029–0.149
*x*_2_	0.100	0.004	0.006	0.09–0.183	0.049	0.001	0.006	0.041–0.058	0.185	0.016	0.004	0.125–0.244

Combined regression variables were analyzed for Flow Rate*x*_1_ and Temperature-*x*_2_, respectively.

^a^Standard-error.

The %EMSβ values will be the response variable for test planning. The response surface methodology (RSM) was applied to transform variables into factors and optimize the experiment^12–14^. The planning results show the importance of optimizing the experiments and reaffirm their statistical importance. The RSM was applied to determine the levels of factors that affect %EMSβ. Table 2 shows the p-value coefficients, which have a significance effect of 0.05 in our predictive model.

The experimental responses encoded by factors × 1 and × 2 can also be represented by general mathematical solutions that locate stationary points, with a p-value of 0.05, such as Eqs. (1–3).

1 $$\% {\text{EMS}}\upbeta {\text{-Leaves}} = \, 0.{499 } + \, 0.0{\text{9 x}}_{{1}} + 0.{1}00{\text{x}}_{{2}}$$

2 $$\% {\text{EMS}}\upbeta {\text{-Stem}} = \, 0.{764 } + \, 0.{\text{181 x}}_{{1}} + 0.0{\text{49x}}_{{2}}$$

3 $$\% {\text{EMS}}\upbeta {\text{-Roots}} = \, 0.{65}0 \, + \, 0.0{\text{89 x}}_{{1}} + 0.{\text{185x}}_{{2}} $$

Equations (1–3) show the extractions of the parts of the plant, offering a unique analysis of each equation associated with its respective graph. Equation (1) shows that the midpoint of %EMSβ—Leaves is 0.499%. The two factors interfere with the extraction efficiency of the β-ecdysone compound. In Eq. (2), the %EMSβ midpoint was the one with the highest value, 0.764, its efficiency can be increased by increasing the temperature and solvent flow. Equation (3) also presents significance between the factors, and its efficiency is also proportional to the increase in flow and temperature.

**Figure 1:**
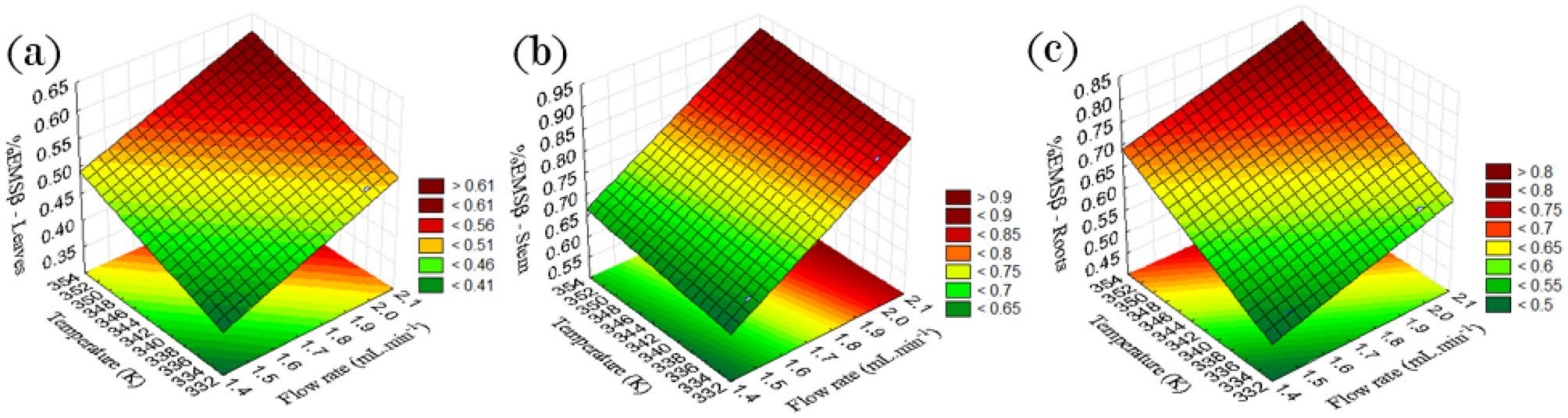
**(a)** Response surface obtained by the Statistica software, for leaf samples; **(b)** stem samples;** (c)** root samples.

**Figure 2:**
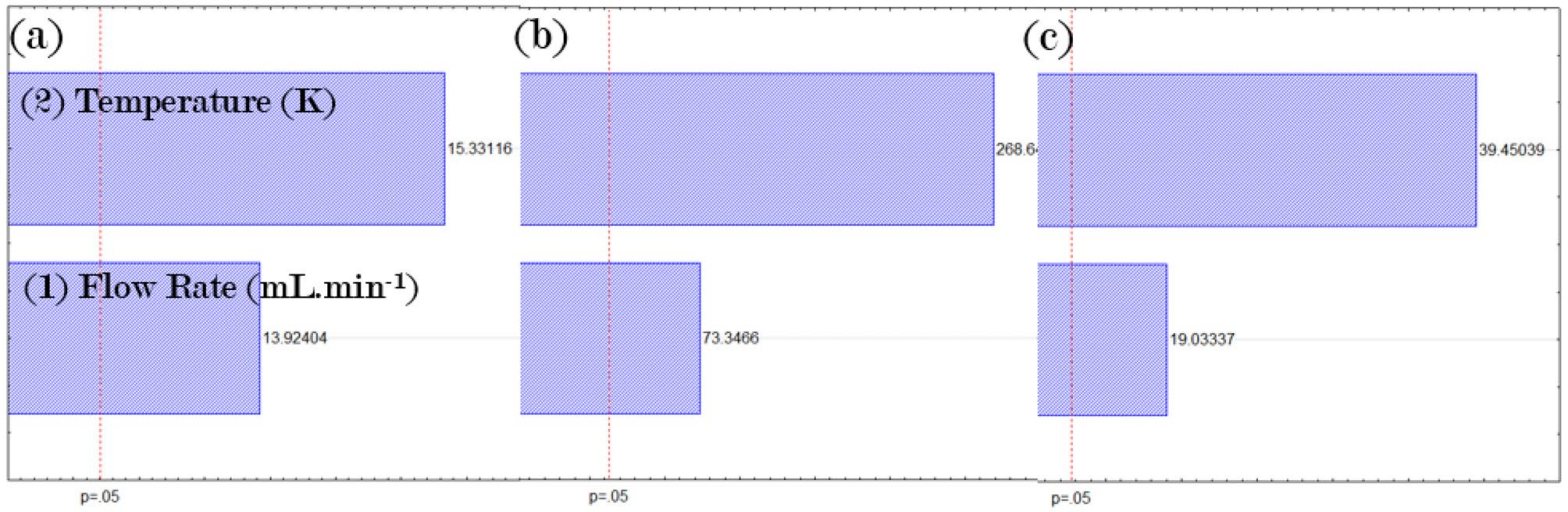
Pareto chart: estimation of the linear effects of the variables. **(a)** Leaf samples,** (b)** stem samples, **(c)** root samples.

The estimated p-value of %EMSβ shows that both factors are significant for planning (Table 2). Therefore, it is necessary to evaluate the optimal condition of the analyzed factors. The extraction yield of β-ecdysone can be shown by observing the RSM graph, as shown in Figs. 1 and 2.

Figure 1 shows that the closer to the stationary dark red region, the higher the %EMSβ. This means that the best region to obtain %EMSβ is between 2.1 and 2.0 solvent flow and 352 (K) to 354 (K). In Fig. 1a–c, it is observed that the increase in yield is not proportional to the increase in solvent flow and temperature. In Fig. 1b, this relationship has a more proportional tendency. In general, the yields obtained at the two temperatures studied were discrepant, and the energy spent on extractions at 2.0 mL min^−1^ 353 K leads to more extractions. As in the study by Chen^15^, which obtained the best process condition using 70% ethanol solvent, retention pressure time of 5 min, extraction temperature of 353 K.

Figure 2 proves that the two linear factors tested in this study are significant. Finally, the data presented demonstrates that the statistical analysis was valid for the extraction PLE for root, stem, and leaves of ginseng and the results obtained by the software were consistent with the extraction performed. The best part of the plant for extracting β-ecdysone is the stem, as it presents the highest yield compared to the other parts of the plant.

### Chromatographic analysis-HPLC

The quantification of 20-Hydroxyecdysterone in optimized samples of PLE extracts (root—T25, stem—T11, and leaf—T1) were carried out by by high-performance liquid chromatography (HPLC). The calibration plot was linearly related to β-ecdysone concentration over the range of 30 mg L^−1^ to 250 mg L^−1^ and the analytical equation is expressed as: Y =  − 479x – 5157 (R = 0.9995)^3^. The limit of detection (LOD) and the limit of quantification (LOQ) values were calculated using as LOD = 3 s/S and LOQ = 10 s/S, where “s” is the standard deviation of y-intercepts (n = 3) and “S” is the slope of the calibration curve^16^. Thus, the LOD and LOQ values are 2 mg L^−1^ and 8 mg L^−1^, respectively^3^.

**Figure 3:**
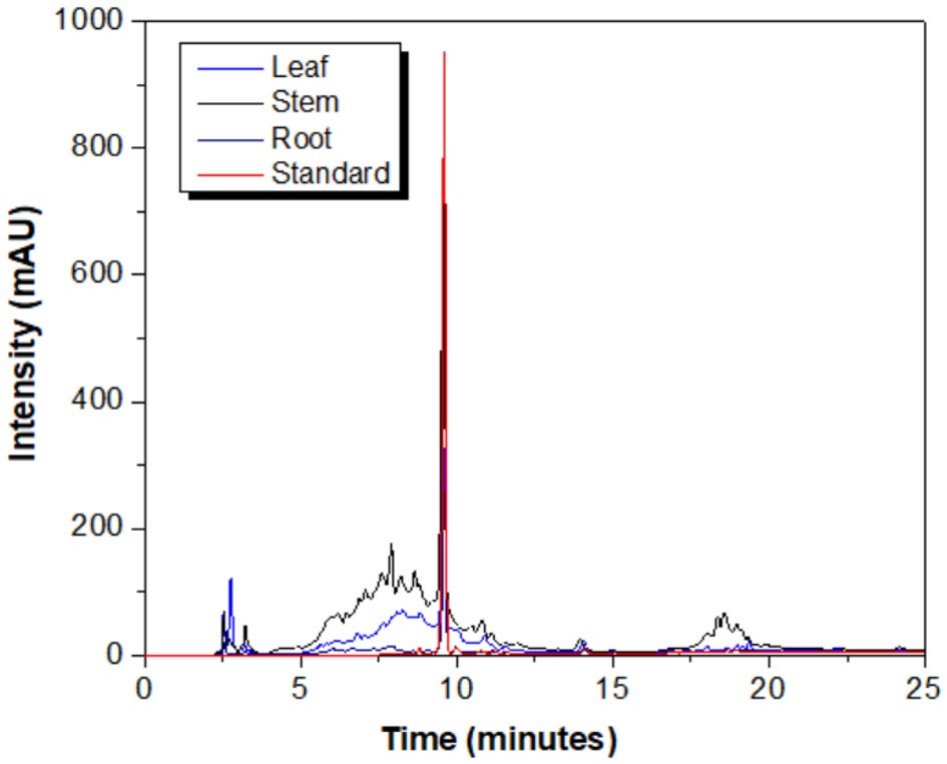
Chromatogram of the leaf extract, stem extract, root extract and β-ecdysone standard.

Figure 3 shows the leaf, stem, and root extracts chromatograms, as well as the beta-ecdysone standard chromatogram.

The results for the quantification of β-ecdysone in 50 mg of the extract for the different samples are showed in Table 3.

**Table 3:** Quantification of β-ecdysone in PLE extracts Ginseng.

Sample	Concentration of β-ecdysone (mg L^−1^)	PLE Extraction (%w/w)
Root	76.53	1.53
Stem	195.86	3.92
Leaf	86.82	1.77

The highest yields (%) found were Stem (3.92), Leaf (1.77), and Root (1.53), respectively. In the root samples, it is observed that there was a lower percentage yield of β-ecdysone than that obtained by Soxhlet extraction (1.63%) reported by^17^ using a 90:10 (v/v) ethanol: water mixture. However, the result was higher than that reported by Vigo, Narita, and Marques, 2004^18^, where the authors used methanol in the Soxhlet extraction and found 1.07% of β-ecdysone. Compared to the study reported by^18^, the result obtained in this study showed 0.69% more β-ecdysone. Vardanega et al.^19^, analyzing the extract of *Pfaffia glomerata* roots obtained by subcritical water extraction (SWE), obtained a yield of 0.70%, and for the aerial parts of the plant, 0.30% of β-ecdysone. This indicates that the PLE extraction can be more efficient to obtain 20E than the classical method.

About the aerial parts of the plant, a study conducted by^20^ used maceration as the extraction method for flower, root, leaves, and stem samples. The HPLC results for the extracts were 0.82%, 0.66%, 0.60%, and 0.24%, respectively. In the case of stem and leaf samples in this study, the yield was higher than 1.17% for the leaf and 3.06% for the stem. In the latter case, the 3.3% yield can be explained due to the initial accumulation of secondary metabolites in the stem, which are later transferred to the roots during their development. According to^21^ reserves are relocated to the roots near their maturation stage. Another contributing factor may have been the conditions applied to the PLE extraction, where the use of a 70/30 (v/v) ethanol: water solution, combined with high pressure, facilitated the rupture of stem cells and subsequent release of metabolites such as β-ecdysone.

Regarding the leaf samples, the different methodologies showed little percentage variation, with PLE extraction being 0.48% higher. This result is significant when compared to the study conducted by^20^ which obtained a yield of 0.60% for their samples.

The presence of β-ecdysone has been reported by different researchers, and the levels vary from accession to accession, where environmental and anthropogenic factors directly interfere with the accumulation of the compound in different parts of the plant.

### Analysis FT-Raman spectroscopy

FT-Raman spectroscopy was carried out to identify molecular structure in the PLE extracts of *P. glomerata* obtained from leaves, roots and stems.

**Figure 4:**
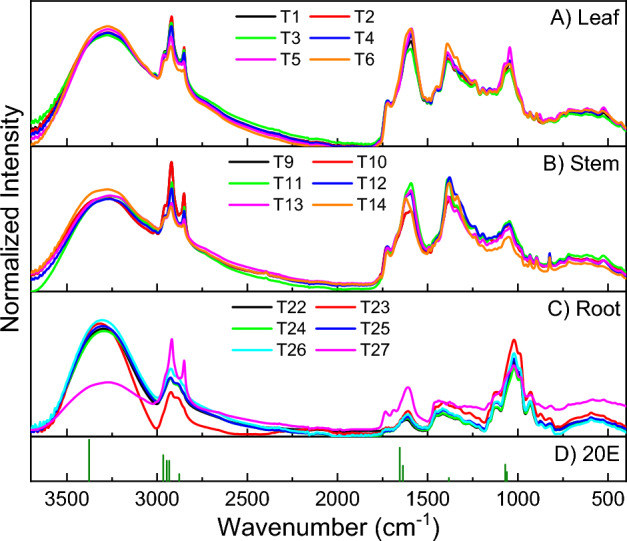
FT-Raman spectra from PLE extracts of leaf, stem, root and 20E samples. Extraction conditions: (**A**) leaf: T1—flow rate 2.0 mL min^−1^ and 80 °C; T2: flow rate 1.5 mL min^−1^ and 80 °C; T4—flow rate 2.0 mL min^−1^ and 60 °C; T5—flow rate 1.5 mL min^−1^ and 60 °C. (**B**) Stem: T10—flow rate 1.5 mL min^−1^ and 80 °C; T11—flow rate 2.0 mL min^−1^ and 80 °C; T12—flow rate 2.0 mL min^−1^ and 60 °C; T13—flow rate 1.5 mL min^−1^ and 60 °C. (**C**) Root: T24—flow rate 1.5 mL min^−1^ and 80 °C; T25—flow rate 2.0 mL min^−1^ and 80 °C; T26: flow rate 1.5 mL min^−1^ and 60 °C; T27—flow rate 2.0 mL min^−1^ and 60 °C. (**D**) β-ecdysone (20E) standard main peaks.

The Fig. 4A–C show the spectra obtained from PLE extraction of leaf, stem and root, in different extraction conditions, which exhibit similar Raman spectral features. A large band of hydroxyl group are present at the region between 3700 and 3000 cm^−1^^22^. Well defined bands of CH3 stretching of aliphatic chains from lipids can be observed in the region 3000–2790 cm^−1^. The presence of the peak at 1730 cm^−1^ was attributed to C=O stretching of esters, while ~ 1600 cm^−1^ to amide I^23^. Between 1500 to 1200 cm^−1^ indicate deformations of HCH and CH_2_OH bonds. Stretching vibrations of C–O bonds, with contributions from C–C bonds of aliphatic chains were associated with spectral region 1130–1015 cm^−1^. Nearby 1050 cm^−1^ is characteristic band of polysaccharides, indicating the existence of amylose and amylopectin^23,24^.

The stem presents a peak at 820 cm^−1^ characteristic of lipids and sulfolipids^23^. In the root spectra the Raman band at 1460 cm^−1^ was observed as characteristic of compounds containing the trace element selenium (Se), such as selenophenes, and it is also characteristic of carbohydrates and lipids^23^. The peak at 930 cm^−1^ coud be associated to esters, carboxylic acids, salts, and complexes of diselenocarbamic acid, selenates, selenites, seleninyl halogenates, amino acids, and disaccharides^23^.

Figure 4D shows most intense Raman bands of 20E. Comparing on PLE extracts of leaf, stem and root spectra with 20E spectrum is possible to note the presence of 20E bands in all spectra. This finding is consistent with the results obtained from HPLC analyses (Table 3).

## Experimental

### Material and methods

#### Sample identification

The root, stem, and leaf samples of *P. glomerata* were obtained through a partnership with the Chico Mendes Institute for Biodiversity Conservation (ICMBio) at number 60553 and access to the genetic material (permission number A7C536D) was granted by the Brazilian National System for the Management of Genetic Heritage and Associated Traditional Knowledge (SISGEN), under current Brazilian biodiversity legislation.

The samples used in the present study are from ginseng producers with a collection site on Jujuí Island, a fluvial archipelago of the Paraná River, geographic coordinates: 23°05′04.54" S, 53°37′30.74" W, 234 m, city of Querência do Norte, Paraná state, Brazil. The ginseng botanical deposit is registered at Herbarium Anchieta-PACA, PACA 118716, located at the Anchietano Research Institute / UNISINOS, municipality of São Leopoldo in the state of Rio Grande do Sul, Brazil.

The presente study utilized the root, stem, and leaf samples of Pfaffia glomerata that were obtained by permission of brazillian Chico Mendes Institute for Biodiversity Conservation (ICMBio) at number 60553 and access to the genetic material (permission number A7C536D) was granted by the Brazilian National System for the Management of Genetic Heritage and Associated Traditional Knowledge (SISGEN), under current Brazilian biodiversity legislation. The ginseng botanical deposit is registered at Herbarium Anchieta-PACA, PACA 118716, located at the Anchietano Research Institute / UNISINOS, municipality of São Leopoldo in the state of Rio Grande do Sul, Brazil. The collection of plant material, comply with relevant institutional, national, and international guidelines and legislation.

### Plant material preparation

The root, stem, and leaves were separated, crushed, and dried in a circulating air oven (Solab brand) at 333 K. The drying time for the stem and leaf samples was 15 h, while the root was 24 h. In total, both reached 10% of humidity determined in the humidity balance (Gehaka brand, model IV 2500). The dry samples were ground in a knife mill (Solab brand, model SL-31) and packed in airtight packages in a freezer at 255 K until the extraction process.

### Experimental planning

The extractions were performed in duplicate based on the full 2^2^ factorial design. Two levels and two variables were investigated for the best operating condition. Four experiments were carried out to analyze the percentage of removal efficiency of the secondary metabolite β-ecdysone (%EMSβ). The model used correlates the flow and temperature variables as a function of the dry extract response variable^12^. According to equation 4.

4 $$y = {\beta }_{0} + {\beta }_{1}{x}_{1}+{\beta }_{2}{x}_{2}+\in$$

The factors were defined as lower (−1), upper (1), to adjust the surface response. In this study, the temperature (×1) and flow rate of the pressurized fluid (×2) were analyzed. The temperature varied from 333 to 353 K and the solution flow rate was 1.5 to 2.0 mL min^−1^^12^. The experimental design used to optimize the %EMSβ response variable. Polynomial equations are obtained by dependence and independence of factors in the response. The data were subjected to analysis of variance (ANOVA), a significance of 5% was defined for the difference between the mean values of the tested parameters^13,14,25^. Table 4 shows the factors and levels of planning.

**Table 4:** Factors and levels that make up the complete factorial design 2^2^ used in the PLE extraction of β-ecdysone from the roots stem, and leaves of *P. glomerata* using 99.5° ethanol and distilled water.

Factor	Units	Variables	
		(−1)	(1)
Temperature	°K	333	353
Flow rate	mL min^−1^	1.5	2.0

### Pressured liquid extraction (PLE)

The conditions for the extraction process were: 5 g of root, stem, and leaf sample separately, constant pressure of 300 Bar, ethanol solvent (70) and water (30) (v/v), temperature of 333 and 353 K and flow rate of 1.5 and 2.0 mL min^−1^. The total extraction kinetics per sample was 60 min for each condition, with the initial 10 min of conditioning the sample with the system (solvent) and the remaining 50 min of effective extraction. For all the runs (Table 4), this procedure was carried out so as not to affect the performance parameters of the extract dry and collection yields^26,27^. The extraction was performed in triplicate. The hydroethanolic extracts were evaporated in an oven with circulation (Solab brand) of air at 333 K. The response variable of the analyzed experimental design will be the dry extract due to the analysis of 20-hydroxyecdysterone. The extracts were determined using an analytical balance (Denver Instrument, model: APX-200).

**Figure 5:**
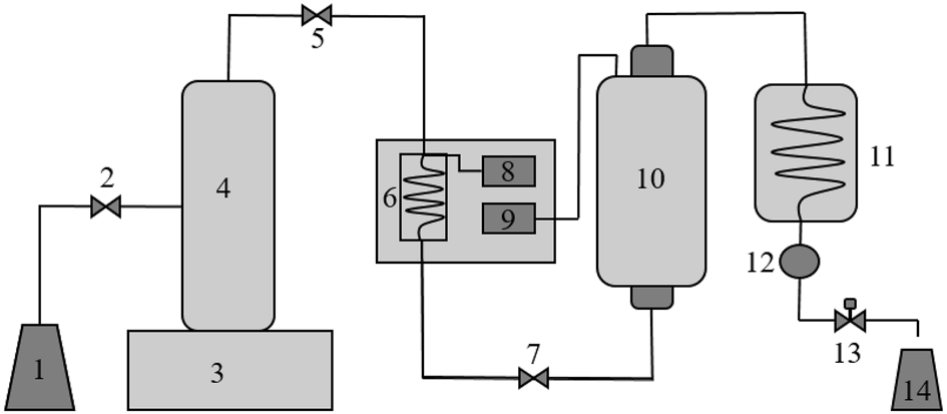
Scheme of the equipment used for the extraction by pressurized liquid (PLE). (1) solvent supply container, (2) needle valve, (3) syringe pump controller, (4) syringe pump, (5) needle valve, (6) preheater, (7) needle valve, (8) preheater temperature controller, (9) extractor container temperature controller, (10) extractor container, (11) cooling system, (12) pressure indicator, (13) back pressure valve, (14) collection container^28^.

The extraction via pressurized liquid was performed by a continuous flow system developed by the Department of Chemical Engineering (DEQ) of the State University of Maringá (UEM), according to the scheme described in Fig. 5.

### Chemical characterization of the PLE extracts

#### High-efficiency liquid chromatography (HPLC)

The 20E quantification was performed according to the methodology adapted by^3^. 20-hydroxyecdysone (beta-ecdysone) (Sigma Aldrich, St. Louis, USA) was used as standard with purity ≥ 93%. Analysis was carried out using Jasco HPLC system, model LC-4000, quaternary pump RHPLC (pressure up to 700 Bar), UV–Vis detector with deuterium lamp and communication module with ChromNav 2.0 software, column C18 (octadecylsilica), with particles of 5.0 µm and dimensions of 250 × 4.6 mm Fortis brand. The separation occurred in a gradient system with different mixtures of methanol/water in the mobile phase. At time 0 to 5 min, the concentration of methanol:water used was from 10:90 (v:v) to 70:30 (v:v). At times 5 to 12 min, the concentration of methanol/water remained at 70:30 (v:v). Into 12 to 15 min the methanol/water concentration ranged from 70:30 (v: v) to 100% methanol. The wavelength was 245 nm and the flow rate was 1 mL/min for a run time of 15 min. The volume of sample injected manually is 20 μL and for each sample three analytical repetitions were performed.

The elaboration of the calibration curve of the β-ecdysone standard different concentrations was used that varied from 30, 60, 100, 150, 200, 250, and 300 mg/L. Standard stock solutions of β-ecdysone in 2 mg/L of methanol HPLC grade (Merck) were used. The retention time of β-ecdysone was 9.6 min.

### FT-Raman spectroscopy

Raman spectra of the samples were recorded in an FT-Raman spectrometer (Bruker, Vertex 70v, Ram II) with Fourier transform (FT-IR) coupled to the Raman scattering detector. The excitation source Nd^3+^-YAG laser, wavelength 1064 nm for and rated power of 500 mW. The germanium detector was kept under liquid nitrogen refrigeration. The data were processed using the OPUS^®^ 6.5 program. There were 500 scans for each spectrum, spectral resolution of 4 cm^−1^, measured between 4000 and 400 cm^−1^. All analyses were performed in duplicate.

## Conclusion

This research provides novel insights into the effectiveness of PLE using environmentally friendly solvents in recovering bioactive compounds from Pfaffia glomerata. The combination of temperature and flow, with constant pressure (300 Bar), time (60 min), and solvent composition (ethanol and distilled water in a 70:30 ratio, v/v), may result in varying amounts of beta-ecdysone depending on the specific plant part under study. The results presented here, to the best of our knowledge, constitute the first published data on the production of PLE extracts with a high concentration of β-ecdysone from the roots, leaves, and stems of *Pfaffia glomerata*. Additionally, the unique potential of Raman spectroscopy for investigating 20E in PLE extracts from *Pfaffia glomerata* is emphasized. Nevertheless, further studies are warranted to comprehensively understand the influence of other variables on the yield of ginseng extracts.

## Data Availability

All data generated or analysed during this study are included in this published article and its supplementary information files.
